# Hepatic Steatosis Index and Chronic Kidney Disease among Middle-Aged Individuals: A Large-Scale Study in Japan

**DOI:** 10.1155/2021/9941834

**Published:** 2021-06-09

**Authors:** Hirotaka Ochiai, Takako Shirasawa, Takahiko Yoshimoto, Satsue Nagahama, Ken Sakamoto, Minami Azuma, Akatsuki Kokaze

**Affiliations:** ^1^Department of Hygiene, Public Health and Preventive Medicine, Showa University School of Medicine, Tokyo, Japan; ^2^All Japan Labor Welfare Foundation, Tokyo, Japan

## Abstract

**Background:**

Though nonalcoholic fatty liver disease (NAFLD) is related to chronic kidney disease (CKD), it is unclear whether the hepatic steatosis index (HSI), a screening tool for NAFLD, is related to CKD. The present study investigated the relationship between HSI and CKD among middle-aged individuals in Japan.

**Methods:**

Subjects were adults (aged 40–64 years) who received an annual health checkup in Japan between April 2013 and March 2014. Height and weight were measured, and venous blood samples were obtained to determine alanine aminotransferase (ALT), aspartate aminotransferase (AST), and creatinine levels. HSI was calculated by the following formula: HSI = 8 × ALT/AST ratio + body mass index (+2, if diabetes; +2, if female). CKD was defined as an estimated glomerular filtration rate < 60 mL/min/1.73 m^2^ and/or urinary protein of ≥ (+). Logistic regression analysis was performed to estimate the odds ratio (OR) and its 95% confidence interval (CI) for CKD.

**Results:**

Data of 94,893 adults were analyzed. Compared with men with an HSI < 30, men with 30 ≤ HSI ≤ 36 (OR: 1.50, 95% CI: 1.40–1.61) and HSI > 36 (OR: 2.14, 95% CI: 1.99–2.31) had significantly higher ORs for CKD. Moreover, there was a significant dose-response relationship between HSI and CKD (*P* for trend < 0.001). Even after adjusting for confounders, the significant results persisted. These findings in men were similar to those in women.

**Conclusions:**

This study showed that the HSI was associated with CKD among middle-aged adults in Japan. Additionally, a dose-response relationship of HSI to CKD was observed. The present study suggested that it might be useful to monitor the HSI among middle-aged individuals to detect CKD at an early stage.

## 1. Introduction

Chronic kidney disease (CKD) is reported to be a risk factor for cardiovascular disease (CVD) and CVD mortality [1, 2]. Moreover, the progression of CKD is inevitable, resulting finally in end-stage renal disease (ESRD) [3]. ESRD substantially increases the risk of death [4]. With an estimated prevalence of CKD at 8–16% worldwide [5], CKD is a global public health problem.

A recent meta-analysis showed that patients with nonalcoholic fatty liver disease (NAFLD) have a higher risk of CKD than those without NAFLD, and the association between NAFLD and CKD is stronger in the Asian population than in the European population [6]. Therefore, those with NAFLD, especially in Asia, must be monitored for the early detection of CKD. Although histologic staging and grading using biopsy are the gold standard for the diagnosis of NAFLD [7], it is not feasible to perform liver biopsy solely to identify patients with NAFLD who are at high risk of developing CKD in the general population.

The hepatic steatosis index (HSI) was reported as a simple, efficient screening tool for NAFLD [8]. If the HSI is associated with CKD, it might be possible to use HSI for the identification of populations at high risk of CKD. Although we previously reported the association between the aspartate aminotransferase (AST) to alanine aminotransferase (ALT) ratio and CKD [9], it is unclear whether HSI including body mass index (BMI), diabetes, and sex in addition to AST and ALT is associated with CKD. Accordingly, this study investigated the relationship between HSI and CKD among middle-aged individuals in Japan.

## 2. Subjects and Methods

### 2.1. Subjects

Subjects in this study were adults aged 40–64 years who received an annual medical examination conducted by the “All Japan Labor Welfare Foundation,” a health service provider in Japan, between April 2013 and March 2014. Of the 310,577 subjects, 310,498 consented to the use of their medical examination data for the present study. Of these participants, the measurement of serum creatinine (SCr) levels was performed for 129,910. Because 35,017 participants with missing data were excluded from the analysis, 94,893 participants were analyzed in this study. The present study was a cross-sectional survey that used the data including the study subjects, and several studies that used the data have been reported [9–15].

Written informed consent for the use of the medical examination data in our study was obtained from each participant. This study protocol was approved by the Medical Ethics Committee of the Showa University School of Medicine (Approval No. 2133) and the Ethics Committee of the All Japan Labor Welfare Foundation (Approval No. 2-1-0003).

### 2.2. Data Collection

Information on the smoking status, alcohol intake, and physical activity was collected from a self-administered questionnaire that is recommended for specific health examinations by the Ministry of Health, Labour, and Welfare.

Height and weight were measured in increments of 0.1 cm and 0.1 kg, respectively. BMI was calculated as weight (kg)/height^2^ (m^2^). The measurement of blood pressure (systolic blood pressure (SBP) and diastolic blood pressure (DBP)) was conducted in the sitting position by an automated machine (HEM-907, Omron, Kyoto, Japan).

Blood samples were drawn and transported to an external laboratory (SRL, Tokyo, Japan). Blood glucose, hemoglobin A1c (HbA1c), high-density lipoprotein cholesterol (HDL-C), low-density lipoprotein cholesterol (LDL-C), triglyceride (TG), ALT, AST, and SCr levels were measured within 24 hours of blood collection. The detail of the measurements was described in our previous studies [9–15]. The estimated glomerular filtration rate (eGFR) was calculated with the following formula: eGFR = 194 × SCr (mg/dL)^−1.094^ × age (years)^−0.287^ (if female × 0.739) [16]. Urine analysis was performed using the dipstick test, and the test results were reported as (-), (±), (+), (++), or (+++).

### 2.3. Definitions

Hypertension was determined as SBP ≥ 140 mmHg, DBP ≥ 90 mmHg, or the use of antihypertensive medication [17]. Dyslipidemia was regarded as LDL − C ≥ 140 mg/dL, HDL − C < 40 mg/dL, TG ≥ 150 mg/dL, or the use of an antidyslipidemic drug [18]. Diabetes was defined as a fasting plasma glucose (≥8 hours after the last caloric intake [19]) ≥126 mg/dL, a random plasma glucose ≥ 200 mg/dL, HbA1c (National Glycohemoglobin Standardization Program) ≥6.5%, or the use of an antidiabetic drug [20].

The HSI was calculated by the following formula: HSI = 8 × ALT/AST ratio + BMI (+2, if diabetes; +2, if female) [8]. HSI was divided into three categories (<30, 30–36, and >36) because HSI < 30 rules out NAFLD and HSI > 36 rules in NAFLD [8, 21]. In accordance with recent studies [22–24], CKD was determined as eGFR < 60 mL/min/1.73 m^2^ and/or urinary protein of ≥ (+).

### 2.4. Statistical Analyses

Data are shown as mean (standard deviation) or *n* (%). The data of SCr, ALT, and AST are presented as medians (25, 75th percentile) because the distributions were highly skewed. Wilcoxon's rank-sum test, unpaired *t*-test, or chi-square test were used for the comparison of characteristics between two groups (the CKD group and the no-CKD group). Logistic regression analysis was employed to estimate the odds ratio (OR) and its 95% confidence interval (CI) for CKD. First, we calculated a crude OR. Second, age, smoking status, alcohol intake, physical activity, hypertension, and dyslipidemia were adjusted for as confounding factors [25–27]. Because diabetes was included in HSI, it was not included as a confounder in the model for examining the relationship between HSI and CKD.

All statistical analyses were conducted using Statistical Analysis System (SAS) software (version 9.4; SAS Institute Inc., Cary, NC, USA), and a *P* value less than 0.05 was regarded as statistically significant.

## 3. Results

The mean age of this study's participants (*n* = 94,893) was 50.6 years. The proportion of men (*n* = 65,850) was 69.4%, while that of women (*n* = 29,043) was 30.6%. The prevalence of CKD overall, in men, and in women was 8.3%, 8.0%, and 9.2%, respectively.

The characteristics of the study participants are shown by sex in Tables 1 and 2. Age and BMI were significantly higher in the CKD group than in the no-CKD group. There were significant differences between the CKD and no-CKD groups in smoking status, alcoholic intake, and physical activity. The proportions of those with hypertension, dyslipidemia, and diabetes were significantly higher in the CKD group than in the no-CKD group. HSI in the CKD group was significantly higher than that in the no-CKD group.

Crude and adjusted ORs for CKD were calculated and reported by sex (Table 3). Compared with men with an HSI < 30, men with 30 ≤ HSI ≤ 36 (OR: 1.50, 95% CI: 1.40–1.61) and HSI > 36 (OR: 2.14, 95% CI: 1.99–2.31) had significantly higher ORs for CKD. These findings in men were similar to those in women (OR: 1.73, 95% CI: 1.58–1.89; OR: 2.62, 95% CI: 2.36–2.92, respectively). In addition, there was a significant dose-response relationship between HSI and CKD in both men and women (*P* for trend < 0.001). Even after adjusting for confounders, these significant results persisted.

Next, participants were divided into four groups according to quartiles of HSI levels, and the association between HSI and CKD was evaluated for the sensitivity analysis (Table 4). A higher level of HSI was associated with a significantly higher OR for CKD among men and women (*P* for trend < 0.001). The results persisted even when the confounding factors were adjusted.

## 4. Discussion

We investigated the relationship of HSI to CKD in middle-aged individuals in Japan. A higher level of HSI was associated with a significantly higher OR for CKD in both men and women. This is the first study to examine the association between HSI and CKD, to the best of our knowledge. However, our results need to be treated with caution.

In this study, the prevalence in men (aged 40–49 and 50–59 years) with eGFR < 60 mL/min/1.73 m^2^ was 2.8% and 6.6%, while that in women was 4.5% and 10.8%, respectively. A previous study conducted in Japan showed that the prevalence among those aged 40–49 and 50–59 years was 4.0% and 7.5% in men, while the prevalence was 2.6% and 6.8% in women, respectively [28]. A recent study reported that the prevalence was 4.3% among men aged 40–49 years and 11.0% among those aged 50-59 years, while the prevalence was 3.1% among women aged 40–49 years and 10.0% among those aged 50-59 years [29]. The difference between our results and those of the other studies might be due to the difference in the data collection periods. Data in this study were collected from 2013 to 2014, while data in the other studies were during 2000–2004 and in 2017. A previous survey performed during 2001–2002 reported that the prevalence of CKD was 13.2% among middle-aged people (mean age: 52.1 years) in Korea [30]. A recent cross-sectional study based on data collected from 2015 to 2016 showed that the prevalence of CKD was 7.3% in the middle-aged Chinese population (aged 40–59 years) [31]. The previous study's prevalence is different from that (8.3%) in our study among individuals aged 40–64 years (mean age, 50.6 years), as the current study is based on the data obtained from April 2013 to March 2014. The reasons for this difference in the prevalence could be owing to differences in age, country, and study period.

In our study, HSI > 36 had a significantly higher OR for CKD than HSI < 30. HSI is reported to be a screening tool for NAFLD [8], and HSI > 36 ruled in NAFLD while HSI < 30 ruled out NAFLD. NAFLD has been shown to be associated with CKD [6]. Furthermore, a recent study reported that CKD is likely to be more common in adults with NAFLD than in those without it [32]. Patients with NAFLD have insulin resistance and hypercoagulability (high fibrinogen, factor VII, and von Willebrand factor levels), which are risk factors for CKD [33]. These studies corroborate our study findings to be reasonable, though the risk factors (insulin resistance and hypercoagulability) were not considered in the present study.

The present study results suggested that it is possible to use HSI for the identification of individuals at high risk of CKD. For instance, it could be effective to use HSI in addition to metabolic syndrome (MS), which was reported to be associated with CKD [34, 35], to identify the population at high risk of CKD. Of 76,920 participants with data on waist circumference and fasting plasma glucose in this study, the proportion of individuals with HSI > 36 and MS defined by the criteria of the committee to evaluate diagnostic standards for metabolic syndrome [36–38] was 8.3%. Furthermore, the proportion was 11.5% among those with HSI > 36 and without MS, while the proportion was 5.1% among those with HSI ≤ 36 and MS. Thus, it might be feasible to target populations (8.3%) with both HSI > 36 and MS compared with those (19.8%) with HSI > 36.

A dose-response relationship between HSI and CKD was observed in the present study. HSI has been shown to be associated with C-reactive protein (CRP) levels [21, 39]. CRP is a very sensitive marker of inflammation [40], which contributes to the initiation and progression of CKD [41]. Previous studies have also shown that CRP is associated with diminished renal filtration [40] and CKD [42]. Because there were no data on CRP in this study, further studies will be needed to elucidate the influence of CRP on the relationship between HSI and CKD.

This study included the large-scale sample (more than 90,000 participants) in Japan, which was the strength of our study. However, a few limitations are present. First, some potential confounding factors that were not controlled for might affect our study findings. For instance, family history of CKD and low birth weight are known risk factors for CKD [43]. Because the information on these factors was unavailable in this study, it is necessary to control for them in future studies. Second, this study included data on middle-aged adults who received an annual health checkup in Japan. Thus, it would be difficult to generalize our study findings to other populations. Finally, this study had a cross-sectional survey design. Therefore, the causal relationship between HSI and CKD could not be established. Cohort studies will be needed to examine the temporal relationship between HSI and CKD.

## 5. Conclusion

The present study showed that the HSI was associated with CKD among middle-aged individuals in Japan. Moreover, the dose-response relationship of HSI with CKD was found. This study's findings suggest that HSI might be useful to detect CKD at an early stage, by the monitoring of HSI among middle-aged populations.

## Figures and Tables

**Table 1:** Characteristics of study participants (men).

	No CKD (*n* = 60,608)	CKD (*n* = 5,242)	*P* value^a^
Age (years)	50.3 (7.0)	54.1 (7.0)	<0.001
BMI (kg/m^2^)	23.6 (3.5)	25.1 (3.9)	<0.001
Smoking status (*n* (%))			
None	21,771 (35.9)	2,298 (43.8)	<0.001
Former	11,817 (19.5)	1,237 (23.6)	
Current	27,020 (44.6)	1,707 (32.6)	
Alcohol intake (*n* (%))			
None	16,058 (26.5)	1,712 (32.7)	<0.001
Sometimes	18,532 (30.6)	1,717 (32.8)	
Everyday	26,018 (42.9)	1,813 (34.6)	
Physical activity (*n* (%))			
<60 min/day	40,365 (66.6)	3,634 (69.3)	<0.001
≥60 min/day	20,243 (33.4)	1,608 (30.7)	
Hypertension (*n* (%))	22,034 (36.4)	2,524 (48.2)	<0.001
Dyslipidemia (*n* (%))	31,969 (52.8)	3,182 (60.7)	<0.001
Diabetes (*n* (%))	4,878 (8.1)	963 (18.4)	<0.001
Creatinine (mg/dL)	0.80 (0.73, 0.90)	1.07 (1.00, 1.13)	<0.001
eGFR (mL/min/1.73 m^2^)	81.4 (13.2)	60.6 (15.8)	<0.001
ALT (U/L)	22 (17, 32)	24 (17, 34)	<0.001
AST (U/L)	22 (19, 27)	23 (19, 29)	<0.001
HSI	32.4 (5.5)	34.2 (5.9)	<0.001

ALT: alanine aminotransferase; AST: aspartate aminotransferase; BMI: body mass index; CKD: chronic kidney disease; eGFR: estimated glomerular filtration rate; HSI: hepatic steatosis index. Values are mean (standard deviation) or median (25th percentile, 75th percentile) except where indicated, *n* (%). ^a^Wilcoxon's rank-sum test, unpaired *t*-test, or chi-squared test.

**Table 2:** Characteristics of study participants (women).

	No CKD (*n* = 26,375)	CKD (*n* = 2,668)	*P* value^a^
Age (years)	50.4 (6.9)	53.4 (6.6)	<0.001
BMI (kg/m^2^)	22.1 (3.7)	23.4 (4.0)	<0.001
Smoking status (*n* (%))			
None	19,805 (75.1)	1,755 (65.8)	<0.001
Former	1,770 (6.7)	324 (12.1)	
Current	4,800 (18.2)	589 (22.1)	
Alcohol intake (*n* (%))			
None	14,318 (54.3)	1,292 (48.4)	<0.001
Sometimes	7,905 (30.0)	776 (29.1)	
Everyday	4,152 (15.7)	600 (22.5)	
Physical activity (*n* (%))			
<60 min/day	18,511 (70.2)	1,809 (67.8)	<0.001
≥60 min/day	7,864 (29.8)	859 (32.2)	
Hypertension (*n* (%))	7,625 (28.9)	982 (36.8)	<0.001
Dyslipidemia (*n* (%))	10,775 (40.9)	1,353 (50.7)	<0.001
Diabetes (*n* (%))	881 (3.3)	191 (7.2)	<0.001
Creatinine (mg/dL)	0.60 (0.56, 0.69)	0.82 (0.80, 0.90)	<0.001
eGFR (mL/min/1.73 m^2^)	81.4 (13.0)	56.5 (11.4)	<0.001
ALT (U/L)	15 (12, 20)	18 (14, 25)	<0.001
AST (U/L)	20 (17, 23)	22 (18, 26)	<0.001
HSI	30.9 (5.0)	32.9 (5.7)	<0.001

ALT: alanine aminotransferase; AST: aspartate aminotransferase; BMI: body mass index; CKD: chronic kidney disease; eGFR: estimated glomerular filtration rate; HSI: hepatic steatosis index. Values are mean (standard deviation) or median (25th percentile, 75th percentile) except where indicated, *n* (%). ^a^Wilcoxon's rank-sum test, unpaired *t*-test, or chi-squared test.

**Table 3:** Association between hepatic steatosis index and chronic kidney disease by sex.

	Total (*N*)	CKD (*n* (%))	Crude	Adjusted
			OR (95% CI)	OR (95% CI)
Men (*n* = 65,850)				
36 < HSI	15,606	1,756 (11.3)	2.14 (1.99-2.31)	2.13 (1.97-2.31)
30 ≤ HSI ≤ 36	26,566	2,164 (8.2)	1.50 (1.40-1.61)	1.40 (1.30-1.51)
HSI < 30	23,678	1,322 (5.6)	1.00	1.00
			*P* for trend < 0.001	*P* for trend < 0.001
Women (*n* = 29,043)				
36 < HSI	4,357	661 (15.2)	2.62 (2.36-2.92)	2.42 (2.17-2.71)
30 ≤ HSI ≤ 36	10,387	1,094 (10.5)	1.73 (1.58-1.89)	1.56 (1.42-1.72)
HSI < 30	14,299	913 (6.4)	1.00	1.00
			*P* for trend < 0.001	*P* for trend < 0.001

CI: confidence interval; CKD: chronic kidney disease; HSI: hepatic steatosis index; OR: odds ratio. Adjusted for age, smoking status, alcohol intake, physical activity, hypertension, and dyslipidemia.

**Table 4:** Odds ratios and their 95% confidence intervals for chronic kidney disease in the group with different levels of hepatic steatosis index by sex.

	Total (*N*)	CKD (*n* (%))	Crude	Adjusted
			OR (95% CI)	OR (95% CI)
Men (*n* = 65,850)				
Q4 (35.8 < HSI)	16,196	1,804 (11.1)	2.33 (2.14-2.54)	2.28 (2.08-2.49)
Q3 (31.8 < HSI ≤ 35.8)	16,243	1,377 (8.5)	1.72 (1.58-1.88)	1.57 (1.43-1.72)
Q2 (28.5 < HSI ≤ 31.8)	16,906	1,219 (7.2)	1.45 (1.32-1.58)	1.36 (1.24-1.49)
Q1 (HSI ≤ 28.5)	16,505	842 (5.1)	1.00	1.00
			*P* for trend < 0.001	*P* for trend < 0.001
Women (*n* = 29,043)				
Q4 (33.5 < HSI)	7,326	1,026 (14.0)	2.62 (2.33-2.95)	2.35 (2.08-2.65)
Q3 (30.1 < HSI ≤ 33.5)	7,101	706 (9.9)	1.78 (1.57-2.01)	1.61 (1.42-1.83)
Q2 (27.6 < HSI ≤ 30.1)	7,289	507 (7.0)	1.20 (1.05-1.37)	1.18 (1.03-1.35)
Q1 (HSI ≤ 27.6)	7,327	429 (5.9)	1.00	1.00
			*P* for trend < 0.001	*P* for trend < 0.001

CI: confidence interval; CKD: chronic kidney disease; HSI: hepatic steatosis index; OR: odds ratio; Q1: quartile 1; Q2: quartile 2; Q3: quartile 3; Q4: quartile 4. Adjusted for age, smoking status, alcohol intake, physical activity, hypertension, and dyslipidemia.

## Data Availability

Data are available on reasonable request and only after approval by the Ethics Committee of the All Japan Labor Welfare Foundation.
